# Reconstruction of complex thoraco-abdominal defects with extended anterolateral thigh flap

**DOI:** 10.4103/0970-0358.73428

**Published:** 2010

**Authors:** Prabha S. Yadav, Quazi G. Ahmad, Vinay Kant Shankhdhar, G. I. Nambi, C. S. Pramesh

**Affiliations:** Plastic & Reconstructive Services, Department of Surgical Oncology, TATA Memorial Hospital, Parel, Mumbai, Maharashtra, India; 1 Division of Thoracic Surgery, Department of Surgical Oncology, TATA Memorial Hospital, Parel, Mumbai, Maharashtra, India

**Keywords:** Thoraco-abdominal defect, microsurgical reconstruction, free anterolateral thigh flap, extended anterolateral thigh flap

## Abstract

**Background:**

The reconstruction of complex thoraco-abdominal defects following tumour ablative procedures has evolved over the years from the use of pedicle flaps to free flaps. The free extended anterolateral thigh flap is a good choice to cover large defects in one stage.

**Materials and Methods:**

From 2004 to 2009, five patients with complex defects of the thoracic and abdominal wall following tumour ablation were reconstructed in one stage and were studied. The commonest tumour was chondrosarcoma. The skeletal component was reconstructed with methylmethacrylate bone cement and polypropylene mesh and the soft tissue with free extended anterolateral thigh flap. The flaps were anastomosed with internal mammary vessels. The donor sites of the flaps were covered with split-skin graft.

**Result:**

All the flaps survived well. One flap required re-exploration for venous congestion and was successfully salvaged. Two flaps had post operative wound infection and were managed conservatively. All flap donor sites developed hyper-pigmentation, contour deformity and cobble stone appearance.

**Conclusion:**

Single-stage reconstruction of the complex defects of the thoraco-abdominal region is feasible with extended anterolateral thigh flap and can be adopted as the first procedure of choice.

## INTRODUCTION

The combined complex defects of the thoracic and abdominal wall are relatively rare. This is because, the defects involving the two regions of the trunk are usually massive and result from either severe trauma, which is more often lethal, and from tumour ablative surgery.[1–4] The options for the reconstruction of such massive defects are pedicle muscle flaps, musculocutaneous flaps and free flaps.[1–4] Depending upon the size and complexity of the defect, one or more flaps are used with synthetic or autologous tissue for skeletal reconstruction.[3–7] The micro-reconstruction can be done in one or two stages based on the availability of microsurgical and intensive care expertise.[3,4,6] The anterolateral thigh flap has become the free flap of choice for soft tissue reconstruction of different regions of the body owing to its versatility.[8,9] From our experience, we opine that thoraco-abdominal defects can be reconstructed in one stage with single large anterolateral thigh free flap. The morbidity associated with the flap donor site is however undeniable and hyperpigmentation, contour deformity and cobble-stone appearance were common.

## MATERIALS AND METHODS

From 2004 to 2009, five male patients [Table 1], who had complex defects of the thoracic and abdominal wall following tumour ablation and were reconstructed in one stage, were included in the study. Post-tumour resections that had isolated defects of the chest or abdomen alone were not included. The patients and their pre-operative investigations were assessed by a joint team of thoracic and plastic surgeons, anesthesiologists and physiotherapists for the pre-, intra- and post-operative care. The oldest patient was 46 years and the youngest was 18 years of age. Four of them had primary tumour and one had second recurrence [Case 4]. Curative resections were done in four patients and palliative procedure was done in one [Case 5]. Three patients had chondrosarcoma, one had Marjolin’s ulcer and one patient with second recurrence had dermatofibrosarcoma. One patient had central defect and the rest had lateral defects. In all cases, a minimum of six ribs were removed and in the patient with central defect, the sternum was also removed. All the defects were reconstructed in one stage.

**Table 1:** Clinical, Pathological and operative details of all patients

*Case no*	*Age*	*Sex*	*Pathology*	*Defect*	*Ribs*	*Sternum*	*Defect size in cm*	*Flap size in cm^2^*	*Diaphragm repair*	*Complication*
1	40	M	Chondrosarcoma	Right lateral	6	NR	32 × 20	640	Primary	Nil
2	30	M	Chondrosarcoma	Central	10	R	35 × 22	770	Mesh	Venous congestion
3	18	M	Chondro sarcoma	Right lateral	8	NR	35 × 18	630	Mesh	Wound infection
4	46	M	Dermato fibro sarcoma	Left lateral	6	NR	30 × 18	364	Primary	Nil
5	18	M	Marjolin’s ulcer	Left lateral	6	NR	32 × 20	504	Primary	Wound infection prolonged ventilator support

R - resected, NR - not resected

Simultaneous two-team approach was adopted to minimize the operating time. The largest of the defect was 35 cm × 22 cm. The skeletal component was reconstructed with methyl methacrylate cement [sternum], polypropylene [Prolene] mesh [ribs, diaphragm and inner abdominal wall] and the soft tissue with free extended anterolateral thigh flap. “Sandwich” technique was used in one patient and the rest were reconstructed with polypropylene mesh. Primary closure of the diaphragm was done in three patients and polypropylene mesh was used in two. Three flaps were harvested from left thigh and two from right. Once the flap size was decided, marking of the perforators was done with hand Doppler and the skin paddle was designed. Four flaps had one Doppler signal and one flap had two [Case 5]. All the flaps had Doppler signal near the midpoint of the line from anterior-superior iliac spine to the superior lateral patella. Intraoperatively, four flaps had musculocutaneous perforators and one [case 5] had both musculo and septocutaneous perforators.[10] All the flaps were elevated with a part of the underlying vastus lateralis muscle. The muscle was included to provide bulk and support the musculocutaneous perforators. The longest flap was 33 cm and the widest flap was 18 cm and the largest of our flaps was measuring 770 cm^2^. The flap elevation was done by standard techniques.[8] The pedicle was dissected till the point of origin from the lateral circumflex artery. After the flap was elevated, the haemostasis of the donor site was done and was covered with split-skin graft from the opposite thigh and the limb was splinted. Before insetting the flap, the defect size was reduced with mobilization of tissues from the sides. The flap vessels were anastomosed with internal mammary vessels due to their proximity to the defect. Suction drains were placed under the flaps without interrupting the pedicle or the anastomosis. Drains were removed once the output was less than 20 ml. To facilitate lung function, chest tube drains were placed and were removed after observing normal lung expansion in post-operative chest X-ray. The patients were monitored in the intensive unit for 24 hours and then shifted to the ward except for one patient [Case 5] who required prolonged ventilator support. Chest physiotherapy was started from the first post-operative day and was continued till the patient was completely ambulant. Flap monitoring was done by sterile needle prick test at 2^nd^ hourly interval for the first 24 hours and 4^th^ hourly till the fourth day and then 8^th^ hourly till discharge.

The patients were nursed in supine or propped up position and gradual ambulation was started after inspecting the skin graft on day five. One flap [Case 2] developed venous congestion on the first post-operative day and was re-explored and successfully salvaged. Intra- and post-operative anticoagulation was used only in this patient [intravenous for four days followed by overlapping of oral and intravenous anticoagulation for next four days and oral alone for next three weeks, which was gradually tapered]. None of the patients had breathing difficulty in the post-operative period owing to reconstruction. The minimum duration of hospital stay was 15 days and maximum was 27 days. The follow-up period ranged from 3 to 36 months and one patient was lost to follow-up after first visit [Case 5]. Apart from this patient, none of the others received post-operative radiotherapy.

## CASE REPORTS

### Case 1

Post-chondrosarcoma excision of the right lateral thoracoabdominal region [Figures 1a–g] of a 40-year-old male presented a defect measuring 32×20 cm exposing the lower lung, liver and intestines. The lower six ribs were removed. The diaphragm was repaired primarily and mesh repair of the chest and inner abdominal layer was done. A right free extended anterolateral thigh flap [29×16 cm] was used for soft tissue cover. The wound healed without complications.

**Figure 1a:**
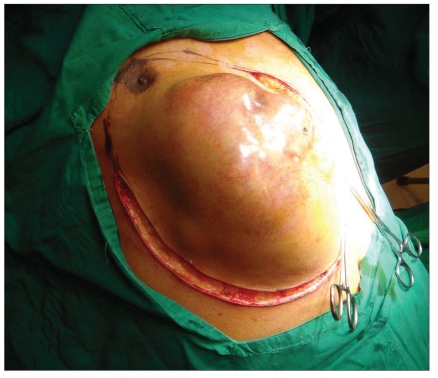
The right chest wall tumour involving the upper abdominal wall

**Figure 1b:**
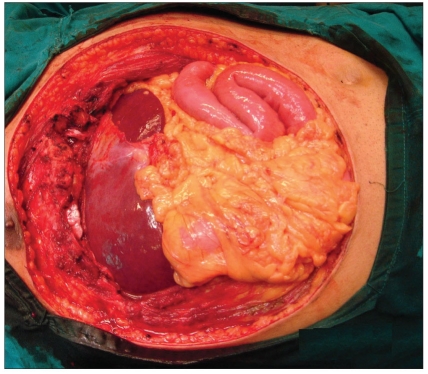
The defect after excision of the tumour, exposing the right lung, diaphragm, liver and coils of intestine

**Figure 1c:**
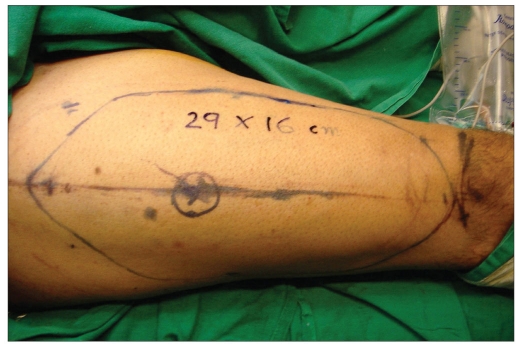
The marking of right antero lateral thigh flap

**Figure 1d:**
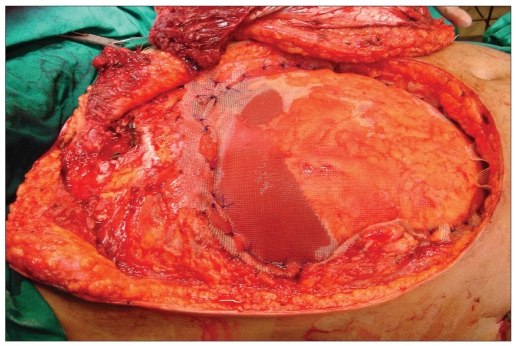
After primary closure of the diaphragm, the right lower chest and deeper layers of the abdomen were repaired with prolene mesh

**Figure 1e:**
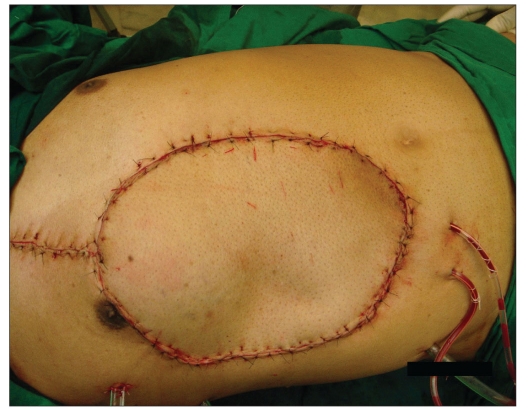
Immediate post op appearance

**Figure 1f:**
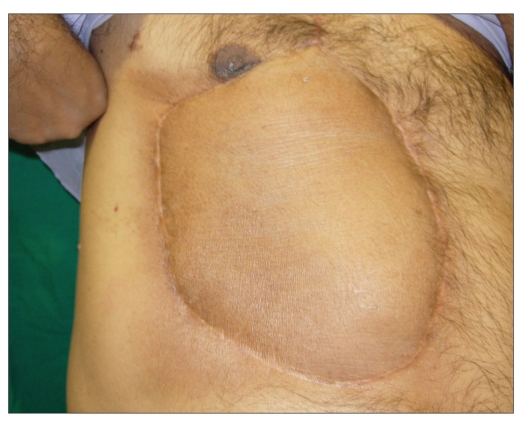
The flap at 12 month followup

**Figure 1g:**
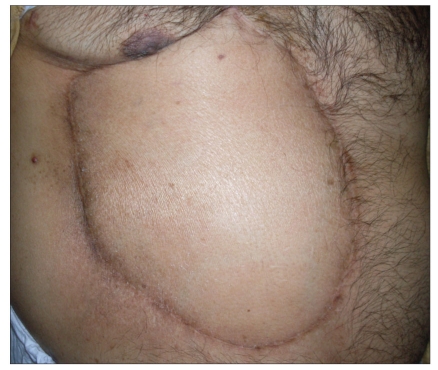
The flap at 36 month followup

### Case 2

Post-chondrosarcoma excision of the lower chest and upper abdomen [Figures 2a–f] of a 30-year-old male presented a defect of 35×22 cm exposing the lungs, the diaphragm, liver, stomach and intestines. The sternum and ten ribs on either side were removed. Polypropylene mesh was used to repair the diaphragm and skeletal component was reconstructed by sandwich technique and the soft tissue cover was provided with left free extended anterolateral thigh flap measuring 25×8 cm. The flap developed venous congestion on first post-operative day and was successfully salvaged after re-exploration. He was maintained on intravenous and later by oral anticoagulation. The wound healed without complications.

**Figure 2a:**
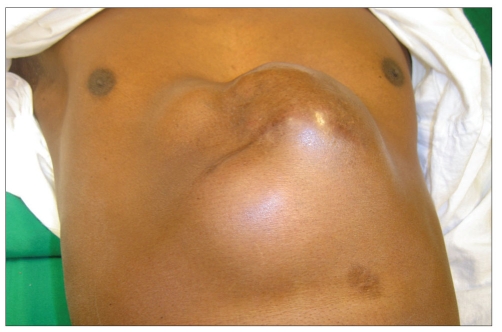
Thoraco abdominal tumour involving the midline - pre op view

**Figure 2b:**
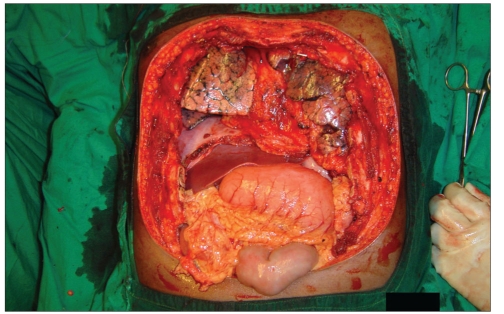
The defect after excision of the sternum and bilateral lower ten ribs, exposing both the lungs, diaphragm, stomach, liver and intestine

**Figure 2c:**
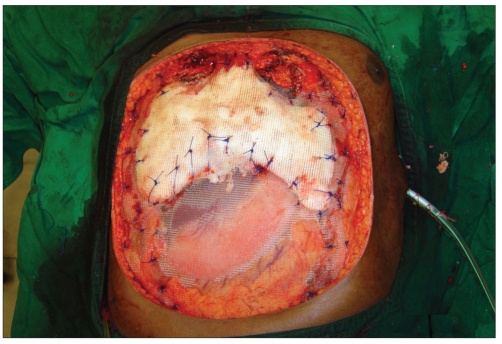
Stabilisation of the chest wall with sandwich technique

**Figure 2d:**
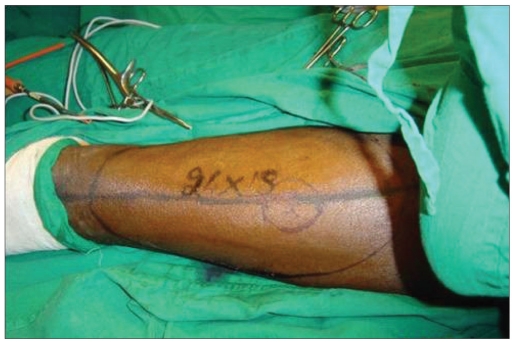
The marking for left antero lateral thigh flap

**Figure 2e:**
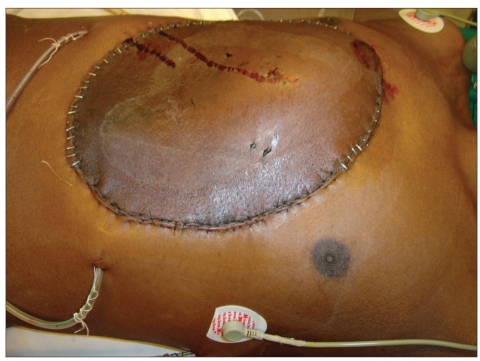
Early post op view showing flap discolouration and dark bleeding on pin prick suggestive of venous congestion

**Figure 2f:**
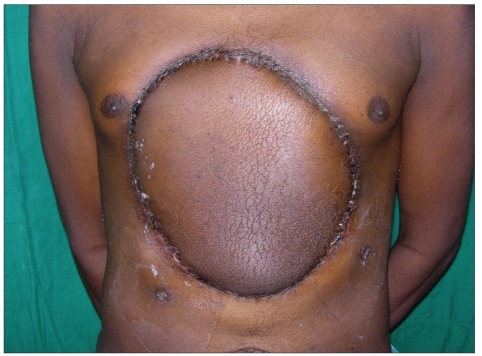
Late post op view - after re-exploration and successful salvage of the flap

## RESULTS

**Figure 3:**
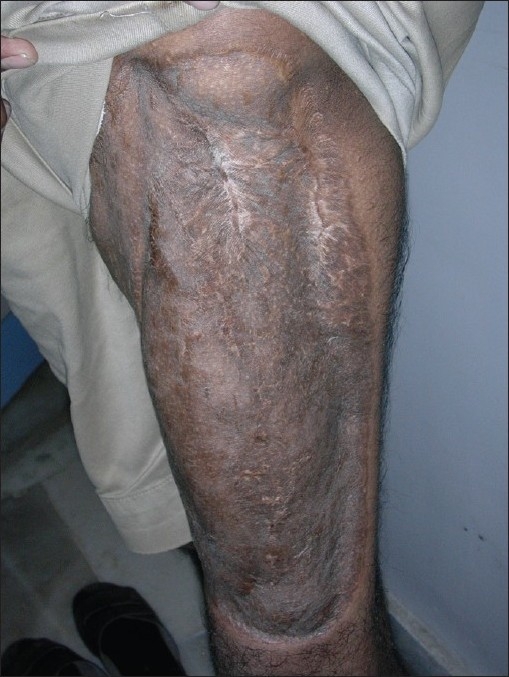
The donor site at 36 months showing hyper pigmentation, contour deformity and cobble stone appearance

Among the five free extended anterolateral thigh flaps, two survived without complications and one flap was re-explored for venous congestion and was successfully salvaged. Two flaps developed suture line infection and were managed conservatively. There was no morbidity regarding the functioning of the intrathoracic or intra-abdominal organs after reconstruction. There were significant morbidities of the donor site in all the patients such as hyperpigmentation, contour deformity and cobblestoning of thigh [Figure 3]. There were no functional deformities of the limb or ventral abdominal hernias.

## DISCUSSION

The aetiology of complex defects of the chest or abdomen alone are well described and may be due to trauma, tumour resection or because of infection[11–14] and a majority of them can be repaired by pedicle flaps[7] or free flaps[6] depending upon the size and complexity of the defect and availability of microsurgical expertise. The combined complex defects of the chest and the abdominal wall are rare. This is because of the fact that trauma causing such a massive defect is usually lethal, and tumours whose resections cause such large defects are rare with very few reports available in the literature.[1–4] The most common tumours are chondrosarcoma, dermatofibrosarcoma, soft tissue sarcoma and desmoid tumour.[1–4] They may arise either from the chest or abdominal wall and involve the other. Surgical resection of these conditions causes significantly larger defects, and reconstructing them is a demanding task for the plastic surgeon. The difficulty is because reconstruction of the chest wall is to be done for both the skeletal and soft tissue components and the abdominal wall for soft tissue components with repair of the diaphragm in between them without compromising the function of the thoracic and abdominal viscera.

Functionally, the reconstruction of the skeletal component of the chest wall is required to stabilize the chest to avoid paradoxical movements and to maintain the post-operative respiratory functions.[3,7] Structurally when more than three ribs on one side or the sternum is removed skeletal reconstruction becomes vital.[11,13] This can be done with autologous tissue such as ribs, bone graft, fascia lata and synthetic materials such as the Polypropylene mesh [Prolene], Polyethylene mesh [Marlex], Polytetrafluoroethylene [Gore Tex] and methyl methacrylate bone cement. Skeletal defects involving the ribs alone can be repaired with one of the synthetic meshes and those with loss of sternum require bone cement to provide stability. Extensive defects involving both the ribs and sternum requires the use of both the synthetic mesh and methyl methacrylate cement as a shield prosthesis, which is also known as sandwich technique.[15] The soft tissue component of the chest wall can be reconstructed with pedicle flaps and free flaps, and they provide the vascularized soft tissue cover to the defect and the prosthetic materials. Depending upon the size and location of the defect, the commonly used pedicle flaps are latissimus dorsi, rectus abdominis, pectoralis major, greater omentum and local perforator-based flaps.[1,2,13] The commonly used free flaps are latissimus dorsi,[13] rectus abdominus,[3,6,13] omentum,[13] tensor fascia lata[3] and anterolateral thigh flap.[16]

The reconstruction of the abdominal wall can be done by primary closure, local flaps, distant flaps and free flaps.[14] The common flaps being latissimus dorsi, rectus abdominis, external oblique, tensor fascia lata[3,14] and anterolateral thigh flap.[17]

The diaphragm that separates the thoracic and the abdominal cavities requires reconstruction in order to maintain the anatomical separation between the two cavities and to maintain the intraabdominal and intrathoracic pressure. Smaller defects are closed primarily while larger defects require reconstruction with synthetic meshes.[18] In this study, in three cases, the diaphragm was repaired primarily in which it was hitched in to the remnant chest wall and in two cases [Cases 2 and 3], polypropylene mesh was used to bridge the defect.

In thoraco-abdominal defects, while the skeletal components can be reconstructed with synthetic materials, the difficulty arises in providing soft tissue cover because, inadequate size or inadequate vascular supply to the soft tissue may compromise the reconstruction with exposure and subsequent infection of the underlying synthetic materials. The disadvantage with the pedicle flaps are that they require change of position of the patient requiring re-painting and re-draping during surgery, and simultaneous two-team-approach may not be possible in every case. This prolongs the operative time and morbidity. Further, pedicle flaps require modification such as extension of the skin paddle or addition of another pedicle flap and therefore increasing the morbidity. These factors can be overcome by single-stage free tissue transfer, which offers simultaneous two-team approach, with reduced operative time and co-morbidity. Depending upon the expertise available, the free tissue transfer can be done in single or two stages with good results. Tukiainen *et al*.[3] used free tensor fascia lata flaps with inclusion of rectus femoris muscle and transverse rectus abdominis myocutaneous flaps for reconstructing thoraco-abdominal defects. Servant *et al*.[4] used free latissimus dorsi myocutaneous flaps to reconstruct similar defects in two stages. Depending upon the availability of recipient vessels, anastomosis are done directly[3] or with the help of interpositional saphenous vein grafts.[3,4] The commonly used recipient vessels for direct anastomosis are inferior epigastric, deep circumflex iliac, superior epigastric and internal mammary vessels.[14] With interpositional vein grafts, the anastomosis can be done with femoral, subclavian or axillary vessels.[3,4]

The anterolateral thigh flap which was described by Song *et al*.[19] and extensively studied by Wei *et al*.[8] has now become the ideal soft tissue flap to cover extensive and complex defects in various regions of the body owing to its versatile nature.[8,9,20] Though there are variations in the type of perforators[10] supplying the skin of the flap, the flaps are reliable when based on either one [septocutaneous or musculocutaneous] or both of them and large skin paddles can be included.[21,22] Since the lateral circumflex femoral artery is also the regional source vessel for muscles in the region such as rectus femoris, vastus lateralis and tensor fasciae latae, one or two of these muscles can be elevated together with their vascular pedicle and the overlying skin, thus making an anterolateral thigh musculocutaneous flap[10] or tensor fascia lata musculocutaneous flap.[3] By using the lateral circumflex femoral artery as the pedicle and incorporating its branches [descending and transverse], large skin flaps can be harvested. Depending upon the surface area of skin added and the pedicle used, these large flaps are also known as hemi-thigh flaps[23,24] [551 cm^2^], giant thigh flaps[25] [105–450 cm^2^], massive or extended anterolateral thigh flaps[21,22] [> 240 cm^2^] and combined anterolateral and anteromedial thigh flaps [500 cm^2^].[26] The hemi-thigh flap[23,24] contains the skin territory of both the anterolateral thigh flap and the tensor fascia lata flap with their corresponding branches [descending and transverse] from the lateral circumflex femoral artery and uses the lateral circumflex artery as the flap pedicle. It includes the deep fascia of the thigh and is thick enough to reconstruct defects that require good soft tissue cover. The giant thigh flap[25] also uses the skin territory of the descending and transverse branches of the lateral circumflex femoral system but it differs from the hemi-thigh flap by its thinness, microdissection and it does not include the deep fascia of the thigh. Further, the giant thigh flap can be elevated on single vascular pedicle [lateral circumflex system] or two vascular pedicles in which the descending and transverse branches are used independently and the lateral circumflex femoral artery is left behind. The combined anterolateral and anteromedial thigh flap has the disadvantage of producing weakness of the knee after flap harvest[26] as both the rectus femoris and vastus lateralis muscles are included. In addition to the larger skin area, the extended anterolateral thigh flap[21,22] includes deep fascia, survives on a single perforator and is not dependent on the addition of tensor fascia lata pedicle. The difference between this flap and the other modifications is the thickness and ease of harvest. As this flap is based on a single perforator, it cannot be thinned as it was found to damage the interlinking vessels between adjacent vascular territories.[22] The dissection is simplified and flap harvest time is reduced as the tensor fascia lata branch is not included.

The average flap size in this study was 581 cm^2^ [range 364–770 cm^2^]. The largest flap in our study was larger than the previous studies[21,22] and two flaps were equal to the largest flaps described previously.[21,22] The addition of a part of the vastus lateralis muscle adds bulk, protects the musculocutaneous perforators, improves local vascularity[26] and possibly reduces the incidence of seroma at the recipient site[21] as none of our cases had seroma at the flap recipient site.

## CONCLUSION

Reconstruction of complex combined defects of the chest and abdominal wall are a rare entity and a significant challenge to the reconstructive surgeon. Such large defects can be managed in one stage with multidisciplinary team approach and close coordination between the team members. Single-stage reconstruction with free tissue transfer offers the advantages of reduced operating time and associated morbidity. Free extended anterolateral thigh flap is an ideal option for one-stage reconstruction of thoraco-abdominal defects, and donor site morbidity though there, should be deemed acceptable when we consider the advantages it offers namely

1. Large area of skin subcutaneous tissue;
2. Does not require thinning;
3. Ease of harvest;
4. Reliable blood supply from a single perforator and
5. Addition of a part of vastus lateralis, which gives bulk, protects the musculocutaneous perforators and reduces the seroma at the recipient site.
